# How neutrophil metabolism affects bacterial killing

**DOI:** 10.1098/rsob.220248

**Published:** 2022-11-23

**Authors:** Juliana E. Toller-Kawahisa, Luke A. J. O'Neill

**Affiliations:** School of Biochemistry and Immunology, Trinity Biomedical Sciences Institute, Trinity College Dublin, Dublin, Ireland

**Keywords:** neutrophil function, glucose metabolism, mitochondrial metabolism, glutamine metabolism, bacterial infection

## Abstract

Neutrophils are front line cells in immunity that quickly recognize and eliminate pathogens, relying mainly on glycolysis to exert their killing functions. Even though investigations into the influence of metabolic pathways in neutrophil function started in the 1930s, the knowledge of how neutrophils metabolically adapt during a bacterial infection remains poorly understood. In this review, we discuss the current knowledge about the metabolic regulation underlying neutrophils response to bacterial infection. Glycogen metabolism has been shown to be important for multiple neutrophil functions. The potential contribution of metabolic pathways other than glycolysis, such as mitochondrial metabolism, for neutrophil function has recently been explored, including fatty acid oxidation in neutrophil differentiation. Complex III in the mitochondria might also control glycolysis via glycerol-3-phosphate oxidation. Future studies should yield new insights into the role of metabolic change in the anti-bacterial response in neutrophils.

## Graphical abstract

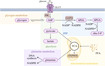

## 1. Introduction

The origins of our understanding of neutrophil metabolism stems from Baldridge & Gerrard [1] who were the first to notice that during phagocytosis, there is an increase in oxygen consumption in leucocytes, which they termed ‘extra respiration of phagocytosis'. In 1959, Sbarra & Karnovsky [2] showed that neutrophils increased their oxygen consumption during the phagocytosis of different types of inert particles but, despite that, phagocytosis occurred equally in aerobic and anaerobic conditions. Importantly, they showed that when these cells were treated with Antimycin A, an inhibitor of respiration, there was no difference in phagocytosis and oxygen consumption, indicating that they were not dependent on mitochondria.

Another important observation was that during phagocytosis there was an increase in glucose utilization and lactate production, showing that the glycolytic pathway was activated and indicating that the energy necessary for phagocytosis was likely to be provided by this pathway. In 1961, Iyer *et al*. [3] found evidence that hydrogen peroxide was being produced during phagocytosis, and they discussed that this could contribute to pathogen elimination. They also showed in more detail the importance of glycolysis and of its metabolites in oxygen consumption by neutrophils. In 1964, Rossi & Zatti [4] demonstrated that nicotinamide adenine dinucleotide phosphate (NADPH) is the primary substrate for the oxidative burst, and Selvaraj & Sbarra [5], in 1966, established that this increase in oxygen consumption, i.e. the respiratory burst, was required for the efficient killing of bacteria by PMNs.

In 1969, McCord & Fridovich [6] showed that hemolysates from bovine blood, which contained neutrophils, were able to convert oxygen into superoxide anion, a powerful oxidation-reduction agent, capable of undergoing either oxidation to $O_{2}^{-}$ or reduction to H_2_O_2_ and liberating energy. Furthermore, Baehner *et al*. [7] were the first group to report the expression of an NADPH oxidase complex in neutrophils. Babior *et al*. [8] found that the superoxide anion was being produced by leucocytes during phagocytosis and hypothesized that superoxide anion could be a possible killing agent in leucocytes. Altogether these findings formed the basis for further investigations in the metabolic contribution to the function of neutrophils. Here, we highlight the current knowledge about the metabolic adaptations underlying neutrophils anti-bacterial response.

## 2. Neutrophil metabolic pathways

**Figure 1.**
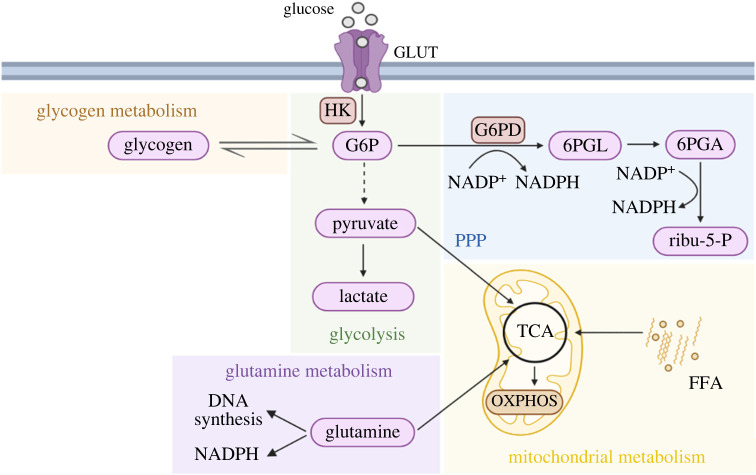
Key metabolic pathways for neutrophil activation. Glycolysis is the major metabolic pathway in neutrophils. Glucose gets inside the cell through GLUT. Once in the cytoplasm, glucose is rapidly phosphorylated to G6P, that is ultimately converted into lactate. G6P can also fuel the PPP to provide NADPH, which is important for some neutrophil functions. Glucose can be stored as glycogen in neutrophil cytoplasm, which is metabolized to glucose to sustain neutrophil function when low glucose is available in the environment. Mitochondrial metabolism is especially important during neutrophils differentiation, as will be discussed further in this review. GLUT, glucose transporter; HK, hexokinase; G6P, glucose-6-phosphate; PPP, pentose phosphate pathway; G6PD, G6P dehydrogenase; 6PGL, 6-phosphogluconolactone; 6PGA, 6-phosphogluconate; ribu-5-P, ribulose-5-phosphate; NADPH, nicotinamide adenine dinucleotide phosphate; TCA, tricarboxylic acid cycle; OXPHOS, oxidative phosphorylation; FFA, free fat acid.

Unlike the respiratory burst, the role of metabolic processes in neutrophil function was neglected. The main metabolic pathway investigated was glycolysis. However, in addition to glucose metabolism, neutrophils can use lipids, proteins and amino acids for energy, and it is now known that additional metabolic pathways are important for neutrophil function, such as the pentose phosphate pathway (PPP), glycogen metabolism, the tricarboxylic acid (TCA) cycle, oxidative phosphorylation (OXPHOS), fatty acid oxidation (FAO) and glutamine metabolism (figure 1).

### 2.1. Glucose metabolism

Neutrophils have a low mitochondrial density [9] and rely mainly on glycolysis for ATP production to exert their function [10,11]. Glucose transport into the neutrophil is dependent on the glucose transporters (GLUT)1, GLUT3 and GLUT4 [12] which ensure the provision of glucose for glycolysis [13]. In addition, neutrophils have a glycogen store, which can serve as a source of glucose [11]. In the cytoplasm, glucose is rapidly phosphorylated by hexokinases to glucose-6-phosphate (G6P) which prevents it from leaving the cell. During glycolysis, G6P is ultimately converted into lactate in aerobic conditions instead of being oxidized in mitochondria through the TCA cycle [11,14]. During phagocytosis, the ATP levels fall from 1.9 nmol/10^6^ cells to 0.8 nmol/10^6^ cells, showing that high levels of ATP are consumed in this process [15].

**Figure 2.**
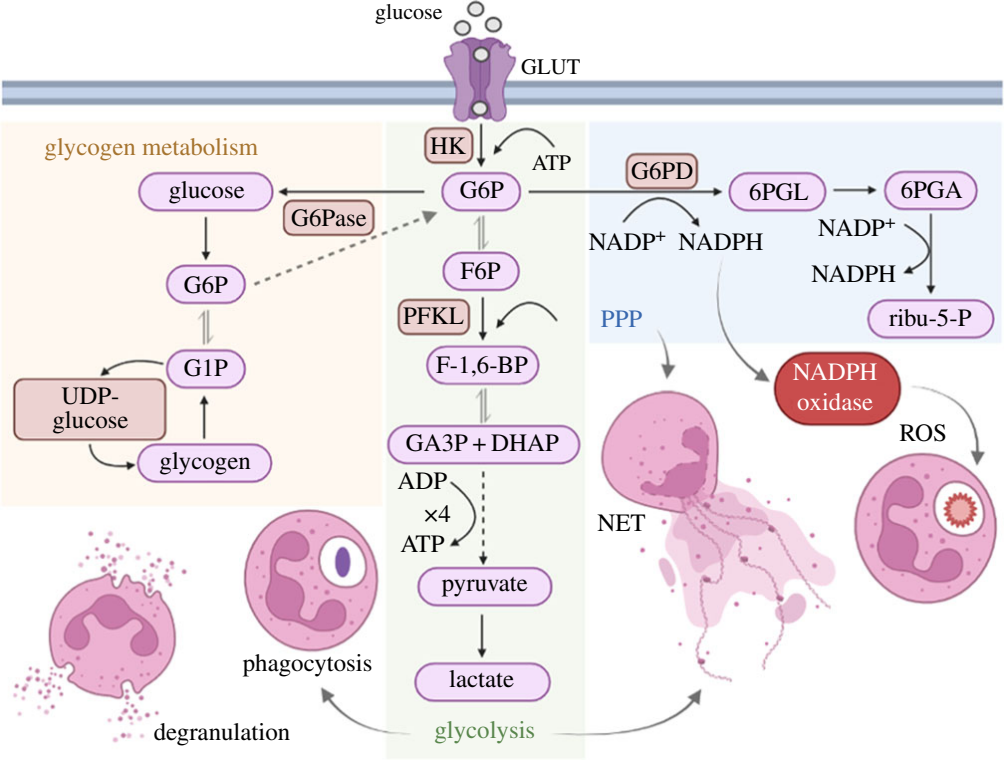
Role of glucose metabolism in neutrophil functions. GLUTs ensure the provision of glucose for glycolysis. Once in the cytoplasm, glucose is rapidly phosphorylated by HK to G6P. During glycolysis, G6P is ultimately converted into lactate producing 4 ATP, of which 2 are consumed during phagocytosis. G6P can also fuel the PPP, that provides NADPH for NADPH oxidase complex, leading to ROS production. PPP activation is also important for NETs formation. Glycogen metabolism provides glucose when low glucose is available in the environment and sustain all neutrophil functions. GLUT, glucose transporter; HK, hexokinase; G6P, glucose-6-phosphate; F6P, fructose-6-phosphate; F-1,6-BP, fructose-1,6-bisphosphate; PFKL, phosphofructokinase; GA3P, glyceraldehyde-3-phosphate; DHAP, dihydroxyacetone-phosphate; G1P, glucose-1-phosphate; PPP, pentose phosphate pathway; G6PD, G6P dehydrogenase; 6PGL, 6-phosphogluconolactone; 6PGA, 6-phosphogluconate; ribu-5-P, ribulose-5-phosphate.

The glycolytic pathway is especially important to fuel the PPP, since it provides NADPH to effectively run the NADPH oxidase complex, leading to ROS production. During the oxidative phase of the PPP, G6P dehydrogenase (G6PD) catalyses the reaction of G6P to 6-phosphogluconolactone (6PGL), producing NADPH. The NADPH donates electrons for the reduction of molecular oxygen to superoxide, which is a starting point to produce secondary ROS [16] (figure 2). Recently, Britt *et al*. [17] showed that neutrophils can rapidly reprogram their metabolism upon stimulation (within 10–30 min), shifting it from glycolysis to PPP to maximize NADPH production. The importance of the PPP can be seen in patients with G6PD deficiency, where neutrophils have decreased NADPH levels and impaired ROS production. They also fail to produce neutrophil extracellular traps (NETs), a web-like structure composed of DNA conjugated with proteins—histones, elastase, myeloperoxidase (MPO), among others—that traps, neutralizes and kills different pathogens [18], which together could account for the recurrent bacterial infections observed in these patients [19].

Some studies showed that the absence of glucose in the media or the inhibition of glycolysis using 2-deoxy-D-glucose (2-DG), a glucose analogue that inhibits glycolysis through competitive binding to glucose hexokinase, impairs ATP, lactate and ROS production, including superoxide [20,21]. As mentioned before, the activation of PPP during glycolysis is especially important for ROS production. The NADPH used by NADPH oxidase complex is produced through PPP activation [16] and the inhibition of G6PD with 6-aminonicotinamide (6-AN) decreases ROS production [22]. Recently, Amara *et al*. [23] showed that the activation of the phosphofructokinase (PFKL), the glycolytic enzyme that converts fructose-6-phosphate (F6P) to fructose-1,6-bisphosphate (F-1,6-BP), impairs ROS production and NETs release by decreasing the activation of PPP and NADPH turnover. In addition, the inhibition of the PPP or protein kinase C (PKC) also impaired PMA-induced NET release, highlighting the importance of NADPH production and NADPH oxidase activation, respectively, for ROS production and thus induction of NETs [24]. In fact, glucose depletion or 2-DG treatment inhibits NETs formation [24,25] (figure 2).

The exact mechanism by which NETs are induced is still being investigated. Rodríguez-Espinosa *et al*. [25] proposed that NETs formation can be metabolically divided in two phases: the first one is independent of glucose and consists of chromatin decondensation, and the second one is dependent of glycolysis activation and leads to NET release. In addition, it is known that some ROS-inducing receptors and kinases such as mitogen-activated protein kinase, extracellular-signal-regulated kinase, PKC, phosphoinositide 3-kinase (PI3K) and protein kinase B (AKT) are linked to NETosis [26–28]. Furthermore, Azzouz *et al*. [29] showed that ROS produced during neutrophil activation induce extensive DNA damage and the subsequent DNA repair pathway activation promotes chromatin decondensation, which leads to NETosis.

Neutrophils contain a large glycogen store in the cytosol, which is especially important when low glucose is available in the environment, as occurs in an inflammatory environment, with G6P being made through glycogenolysis [11]. Recently, Sadiku *et al*. [30] showed that in neutrophils the failure to replenish or access the glycogen stores results in metabolic exhaustion and dysfunctional neutrophil responses. Glycogen and glucose homeostasis is regulated by the glucose-6-phosphatase (G6Pase) complex, localized in the endoplasmic reticulum membrane. This complex hydrolyses G6P to glucose during gluconeogenesis [31]. Defects in G6P/glucose cycling between ER and cytoplasm in neutrophils leads to glycogen storage disease type Ib (GSD-Ib), characterized by impaired glucose homeostasis and neutropenia, in addition to decreased G6P, NADPH, lactate and ATP levels, impaired ROS production and chemotaxis [32], which highlight the importance of glycogenolysis for neutrophil functions (figure 2).

Neutrophil phagocytic function relies mainly on glycolysis, as it is insensitive to mitochondrial respiratory chain inhibitors such as potassium cyanide (inhibits Complex IV) and Antimycin A (inhibits Complex III) but is blocked when glycolysis inhibitors are used [2]. Phagocytosis increases in the presence of glucose and is almost completely impaired when neutrophils were treated with 2-DG, which is accompanied by a drop in ATP levels [33]. Glycolysis activation is also important for degranulation, as treatment with 2-DG decreased lysozyme [34] and MPO [35] release from granules. However, degranulation occurred in glucose-free media and the effect of 2-DG was only seen in the absence of glucose, which may indicate that glycogen metabolism could be more important for this function.

Boxer *et al*. [33] showed that neutrophils treated with 2-DG had impaired phagocytosis of *Staphylococcus aureus* and *Streptococcus pneumoniae*. Silva & Jain [36] treated neutrophils with sodium fluoride (NaF), an inhibitor of the glycolytic enzyme enolase, incubated them with *Escherichia coli*, *S. aureus* or *Streptococcus agalactiae*, and observed that the phagocytic capacity was decreased only for *E. coli*. They also evaluated ROS production and found that when neutrophils were stimulated with *S. aureus*—but not with *E. coli* and *S. agalactiae*—the treatment with NaF decreased ROS production. Recently, Fan *et al*. [37] found that neutrophils treated with 2-DG and incubated with *S. pneumoniae* had impaired phagocytosis and bacterial killing. The reason for different neutrophils' response depending on the bacterium type is unknown but could be due to the sensing of different PAMPs (Pathogen-associated molecular pattern) or the presence of different toxins. This interesting question requires further investigation.

The clinical relevance of the influence of these changes in glucose metabolism in neutrophils' killing capacity can be seen in some pathological conditions. Patients with G6PD deficiency have more prevalence of sepsis, which was associated with impaired neutrophil function [38]. In addition, G6PD deficiency leads to recurrent bacterial infection and impaired ROS production and NETs release [19]. Controversially, other groups failed to show that G6PD deficiency can impair neutrophil function [39,40], which could be due to daily fluctuations in NADPH production [40]. Furthermore, patients with GSD-Ib have neutrophil dysfunction [41] and are prone to frequent infectious diseases [42,43]. In a single case study, neutrophils from a GSD-Ib patient had decreased phagocytosis and killing capacity to *S. aureus*, *E. coli* and *P. gengivalis* [44]. *Diabetes mellitus*, a clinical syndrome associated with the deficiency of insulin secretion or action, is among the known diseases that undermine host defences and increases the susceptibility to bacterial infections mainly through modulation of the immune system [45]. The hyperglycaemic environment blocks G6PD, increasing neutrophil apoptosis and chemotaxis. In addition, it increases intracellular glucose levels, that uses NADPH as a cofactor to be metabolized and prevents the regeneration of molecules that play a key role in antioxidant mechanisms [46]. Some studies showed that the phagocytosis and killing capacity of neutrophils from diabetic patients were reduced when they were exposed to opsonized *S. aureus* [47,48] or pneumococcus [49]. This could be due to a decrease in ROS production [50] and NETs formation [51], but there are conflicting results in the literature. More studies must be done to determine the exact genetic and metabolic mechanisms by which G6PD deficiency, GSD-Ib and diabetes affect neutrophil functions during bacterial infection.

### 2.2. Mitochondrial metabolism

In neutrophils, the main role of the mitochondria is to initiate apoptosis if damage, as the treatment with FCCP or oligomycin, that depletes mitochondrial membrane potential and inhibits ATP synthesis respectively, has no impact on ROS production, phagocytosis and ATP production. However, it induces the loss of the mitochondrial membrane potential, which precedes the appearance of morphological indicators of apoptosis, with the cells adopting a more rounded morphology [9]. Indeed, the basal oxygen consumption rate and ATP generation in neutrophils are unresponsive to mitochondrial respiratory inhibitors [52,53]. Despite that, it is known that mitochondrial metabolism is especially important for neutrophil differentiation in the bone marrow.

**Figure 3.**
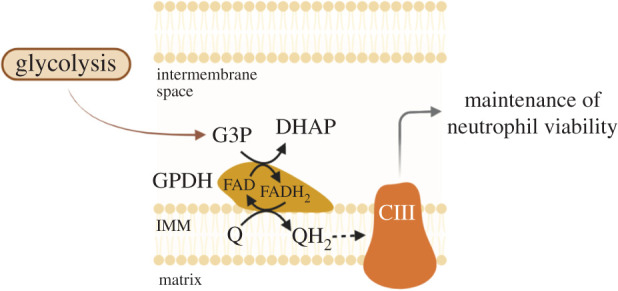
The glycerol-3-phosphate shuttle maintains mitochondrial membrane electrochemical potential in neutrophils. G3P is produced during glycolysis and can diffuse into the mitochondria, where it is re-oxidized to DHAP on the intermembrane space by GPDH. The electrons from G3P are transferred to complex III of the respiratory chain via ubiquinol, maintaining the membrane electrochemical potential. G3P, glycerol-3-phosphate; DHAP, dihydroxyacetone-phosphate; GPDH, glycerol-3-phosphate dehydrogenase; IMM, inner mitochondrial membrane; CIII, complex III; FAD, flavin adenine dinucleotide.

During neutrophil differentiation, their metabolism is shifted from OXPHOS to glycolysis with loss of cytochrome c expression, a vital component of the electron transport chain [53]. OXPHOS is an important mitochondrial process that links the TCA cycle to the production of ATP and creates a membrane electrochemical potential (DYm). The DYm is generated via the coupling of electron transfer and H^+^ pumping via four mitochondrial respiratory chain complexes (CI, CII, CIII and CIV) in the mitochondrial inner membrane. These complexes convert energy into ATP and can form supercomplexes that contain several copies of CI, CIII, and CIV within one respiratory chain, which ultimately creates an increased membrane potential and produces more ATP [14,54]. However, circulating neutrophils lack supercomplexes and, for this reason, they do not couple DYm to efficient respiration and ATP synthesis. In neutrophils, the DYm is maintained mainly through the transfer of electrons from glycerol-3-phosphate (G3P), as the DYm generated with glycerol phosphate as a substrate was higher than with the complex I substrates glutamate/malate, the complex II substrate succinate or in the absence of any substrate. G3P is produced during glycolysis and can diffuse into the mitochondria, where it is re-oxidized to dihydroxyacetone-phosphate (DHAP) on the outer surface of the inner mitochondrial membrane and the electrons from G3P are subsequently transferred to complex III of the respiratory chain via ubiquinol. In addition, the inhibition of CIII increases lactate production from G3P. In this way, in neutrophils the mitochondria play an important role in the regulation of aerobic glycolysis but not ATP production [55]. A decrease in mitochondria (specially in Complex III) will therefore boost glycolysis in neutrophils (figure 3).

**Figure 4.**
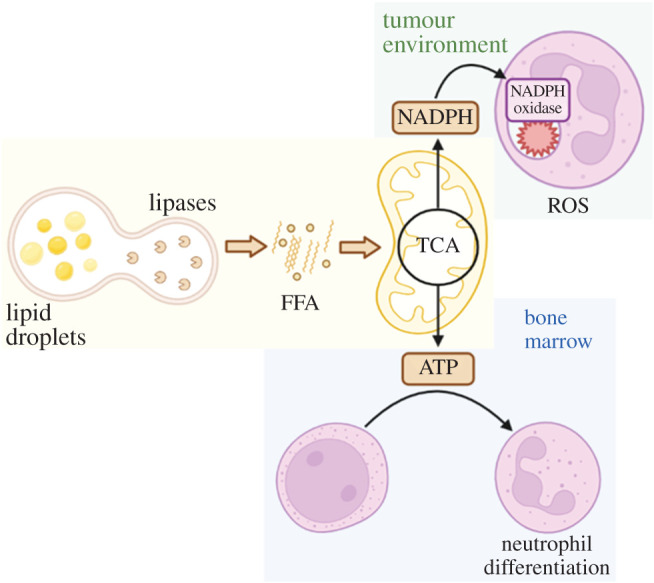
FAO is important for neutrophil differentiation in the bone marrow and ROS production in the tumour environment. Lipid droplets are degraded into FFA during autophagy to provide energy for neutrophil differentiation in the bone marrow. In parallel, tumour-elicited neutrophils can maintain ROS production in a low-glucose environment by producing NADPH via an alternative fatty acid-dependent mitochondrial source. FFA, free fat acids; TCA, tricarboxylic acid cycle; NADPH, nicotinamide adenine dinucleotide phosphate; ROS, reactive oxygen species.

Another important mitochondrial aerobic process to produce ATP is FAO. Briefly, fatty acids enter the mitochondria through an acetylcarnitine transporter after being metabolized into fatty acyl-CoA esters, for subsequent oxidation. At the end of this process, two acetyl-CoA molecules will remain and then enter the TCA cycle and the OXPHOS system which will generate ATP. In neutrophils, it has been shown that FAO is especially important during their differentiation, where autophagy plays a role providing free fatty acids via lipid droplet degradation to maintain energetic-metabolic balance. This FAO-OXPHOS pathway controlled by autophagy seems critical to supply ATP for the energy-demanding process of differentiation [56]. In addition, FAO seems important for neutrophil functions in the tumour environment, where low glucose is available [57]. Despite it being already known that in neutrophils the mitochondria do not contribute to ROS production [9], Rice *et al*. [57] demonstrated that the tumour environment elicits the c-Kit signalling in neutrophils, driving an oxidative phenotype with enhanced mitochondrial function. These neutrophils can maintain ROS production by increasing NADPH levels produced through fatty acid-dependent mitochondrial function, indicating that fatty acid utilization is required for neutrophil function when glucose is limited [57] (figure 4).

Regarding NETs, Rodríguez-Espinosa *et al*. [29] showed that the inhibition of ATP synthase had a small effect and the addition of pyruvate in the culture media had no effect in NETs formation, demonstrating that neutrophils do not rely on mitochondrial function for NETs release. Another interesting observation is that ATP increases NETs release in a process dependent on the ATP channel Pannexin 1 (PANX1) [58]. Alárcon *et al*. [59] showed that non-esterified fatty acids induce NETs formation and ATP release via PANX1 and activation of the ATP receptor P2X1 and that NET formation induced by non-esterified fatty acids is partially dependent of FAO.

Mitochondria would be expected to be less involved in bioenergetics in neutrophils, because in inflammatory environments during an infection, there is a decrease in the oxygen available and most eukaryotic cells shift their primary metabolic strategy from predominantly mitochondrial respiration towards increased glycolysis to maintain ATP levels. This metabolic switch can be regulated by the hypoxia inducible transcription factor-1 (HIF-1), a heterodimer composed by HIF-1*α* (transcription-induced nuclear protein) and HIF-1*β* (constitutive) that has target genes involved in hypoxia adaptation, inflammation development and enzymes involved in the glycolytic pathway [60]. Sadiku *et al*. [61] demonstrated that in myeloid cells the absence of Phd2, a prolyl hydroxylase enzyme that sets the basal level of HIF-1*α* in normoxia, resulted in an increased inflammatory response to *S. pneumoniae* in the lung, with increased neutrophil migration, functional capacity and survival. In addition, Phd2-deficient neutrophils had an increased glycolytic flux and glycogen. Hypoxia can also affect the response of neutrophils to *Mycobacterium tuberculosis*, as seem by decreased NETs formation, but does not affect matrix metalloproteinases secretion and phagocytic capacity [62]. HIF-1*α* might therefore be an important player in neutrophils activation.

### 2.3. Glutamine metabolism

Glutamine is one of the most abundant amino acids in the body and is important for ATP production, nucleic acid synthesis and immune function. It is known that glutamine has no effect on phagocytosis but increases bactericidal activity in neutrophils [63], as well as superoxide anion production and expression of NADPH oxidase subunits [64] and ROS production [65]. In stressed rats, oral glutamine administration increased ROS production by neutrophils [66]. In addition, a study with male judoists that ingested 3000 mg of glutamine per day for two weeks showed that neutrophils produced more ROS compared to placebo [67]. *In vitro*, Castell *et al*. [65] showed that glutamine supplementation increases ROS production in response to the neutrophil activators PMA and fMLP. Regarding NETs, the absence of glutamine diminished but did not inhibit their formation [29]. Recently, Sadiku *et al*. [30] showed that the Gram-negative bacterial cell wall component LPS increases glutamine conversion into glutamate, indicating that glutamine is consumed during neutrophil activation. In addition, they demonstrated that glutamine can be used for the generation of glycolytic intermediates, which could provide an alternative mechanism for neutrophils to meet their energetic demands in low-glucose environments and allow them to generate NADPH for ROS generation.

## 3. Conclusion

In the past few years, the immunometabolism field has attracted much attention. It is now clear that neutrophils mainly rely on the glycolytic pathway to fulfil their metabolic needs in the induction of phagocytosis and NETs formation. The PPP, which is an off-shoot of glycolysis, is needed for NADPH production for the respiratory burst. Broadly speaking, mitochondria play a limited role in neutrophil function, although they can be deployed to oxidize fatty acids should glucose levels be low (e.g. in tumours), or during neutrophil differentiation. Further studies on the role of mitochondria in neutrophils are warranted. Metabolic adaptation could be particularly important for neutrophils because they are exposed to different micro-environments where the availability of nutrients can be scarce. Further investigations are needed to fully understand how metabolism influences the functional activity to bacteria and how metabolic changes in the micro-environment can influence the function of these important front-line cells. The resulting new insights might contribute to new therapeutic strategies during bacterial infection.

## Data Availability

This article has no additional data.
